# Age in relation to comorbidity and outcome in patients with high-risk TIA or minor ischemic stroke: A Swedish national observational study

**DOI:** 10.1177/2396987320975980

**Published:** 2020-12-13

**Authors:** Oskar Fasth, Eva Lesén, Peter Appelros, Bahman Farahmand, Jonatan Hedberg, Per Ladenvall, Carl Mellström, Signild Åsberg

**Affiliations:** 1 Department of Neuroscience, Uppsala University, Uppsala, Sweden; 2 AstraZeneca AB, Göteborg, Sweden; 3 University Health Care Research Center, Örebro University, Örebro, Sweden; 4 Epi-Consultant, Stockholm, Sweden

**Keywords:** Ischemic attack, transient, stroke, epidemiology, mortality, registries, platelet aggregation inhibitors, transient ischemic attack (TIA), ischemic stroke, epidemiology, mortality/survival, secondary prevention

## Abstract

**Introduction:**

Recent trials report positive results for preventing vascular events with dual antiplatelet therapy (DAPT) in patients with high-risk TIA or minor ischemic stroke. We aimed to investigate this population regarding influence of age on vascular risk factors, hospital stay and mortality.

**Patients and methods:**

Data on patients aged 40–100 years with TIA or ischemic stroke in the Swedish Stroke Register during 2012–13 were linked with national registers. To identify patients with high-risk TIA (ABCD^2^ ≥6) or minor ischemic stroke (NIHSS ≤5) eligible for DAPT, we excluded patients with atrial fibrillation, anticoagulant use, prior major bleeding, or unknown stroke severity.

**Findings:**

We identified 10,053 potential DAPT-candidates (mean age 72.6 years, 45.2% female, 16.4% with TIA). With advancing age, most vascular risk factors increased. Antiplatelet treatment increased from 31.9% before the event to 95.5% after discharge. Within 1 year following index event, the proportion of patients with ≥1 re-admission increased with age (29.2% in 40–64 year-olds; 47.2% in 85–100 year-olds). All-cause death per 100 person-years was 6.9 (95% CI 6.4–7.4) within 1 year, and highest in the first 30 days (15.2; 95% CI 12.8–18.2). For each year of increased age, the risk of death increased with 3.5% (p = 0.128) in patients 40–64 years and with 11.8% (p < 0.001) in those ≥85 years.

**Conclusions:**

While in theory representing a subset of patients with mild injury, our observational study highlights substantial use of health-care resources and high mortality rates among patients with high-risk TIA or minor ischemic stroke assumed eligible for DAPT.

## Introduction

Stroke is a leading cause of disability and death worldwide, with an estimated yearly cost of €60 billion in Europe (2017).^1^ Despite a decrease in the age-standardized incidence over time, the overall burden of stroke remains high as the population ages.^2^

Cerebrovascular ischemic events, such as transient ischemic attack (TIA) or acute ischemic stroke (AIS), can have a wide range of presentations, from transient symptoms to large infarctions resulting in major disability and/or death. Outcome after TIA or stroke is correlated to its initial severity and by underlying comorbidities, which in turn are heavily influenced by age.^3,4^ Stroke patients with high-risk comorbidities are also demonstrated to have increased re-admission rates.^5^

After non-cardioembolic TIA or AIS, single antiplatelet therapy has, until recently, been the mainstay for secondary prevention.^6–8^ For patients with high-risk TIA and minor AIS, recent trials report positive results for dual antiplatelet therapy (DAPT) with aspirin and P2Y_12_-inhibitors,^9–11^ and short-term DAPT with aspirin and clopidogrel is now recommended in guidelines.^12,13^ DAPT with ticagrelor and aspirin vs. aspirin alone during the first 30 days is investigated in the recently concluded Acute Stroke or Transient Ischemic Attack Treated with Ticagrelor and Aspirin for Prevention of Stroke and Death (THALES) trial, which reports a reduction of its primary composite endpoint of stroke and death.^11^ Large randomized clinical trials provide the most valid evidence for medical decision-making, but their results can lack generalizability beyond the studied population. Despite increased evidence and stronger recommendations for DAPT in secondary stroke prevention, limited real-world data on the targeted population are so far presented.^7,14,15^

Our overall aim was to increase understanding of patients who, given current guidelines and recent studies, might be considered for treatment with P2Y_12_-inhibitor-based DAPT. Our objectives were to investigate (1) the influence of age on prevalence of risk factors, antiplatelet therapy, all-cause hospital re-admissions, and mortality in patients with non-cardioembolic high-risk TIA or minor AIS; and (2) the relationship between post-stroke disability and re-admissions among patients with non-cardioembolic minor AIS.

## Methods

This observational cohort study used a linked dataset, which is previously described in detail.^15^ In brief, data on all patients registered in the Swedish Stroke Register (Riksstroke) during calendar years 2012–2013 were linked with 3 nationwide healthcare registries: the National Patient Register (NPR), the Prescribed Drug Register (PDR), and the Cause of Death Register (CDR), using the patients’ unique personal identification number. All analyses were performed in agreement with privacy legislation in Sweden. The Swedish National Board of Health and Welfare approved and performed the linkage of data. Uppsala University Hospital, Sweden managed the linked database thereafter.

### Study population

The study population was selected to match, as closely as possible, previous DAPT-trials on TIA/stroke, with the most recent trial (THALES) as reference.^11^ Patients were included if registered in Riksstroke with a diagnosis of high-risk (defined by ABCD^2^ score ≥ 6) TIA or minor (National Institutes of Health Stroke Scale, NIHSS score ≤5) AIS, and aged 40–100 years. Exclusion criteria were: cardioembolic etiology (defined as prior diagnosis of atrial fibrillation or use of anticoagulants 6 months before index), history of intracranial hemorrhage, gastrointestinal bleeding, bleeding diathesis, coagulation disorders, renal dialysis, or missing ABCD^2^/NIHSS scores (Supplemental Material I).

### Variables and definitions

Vascular risk factors, i.e. previous TIA or stroke, hypertension, diabetes mellitus, ischemic heart disease, cancer, and active smoking, were identified via Riksstroke, NPR, and PDR (Supplemental Material II). Since the clinical definition of hypertension differs for TIA and stroke in Riksstroke, we defined hypertension as purchase of ≥2 antihypertensive drug classes before index admission, a previously validated definition (the positive predictive value to predict hypertension was 80.0% and the specificity was 94.7%).^16^ Riksstroke was the source of dates for symptom onset (index event), admission, and discharge, in addition to risk and severity scores at index hospitalization. Number of all-cause hospital re-admissions (acute and planned) after index discharge were collected from NPR. A wash-out period of 1 day since the most recent hospital discharge was applied to minimize misclassification due to between- and in-hospital transfers. For AIS patients, Riksstroke provided information on pre-admission level of activities of daily living (ADL), discharge disposition, and post-stroke disability through questionnaires to patients 3 months after stroke.

The CDR was used for date of death and PDR for medications. Use *before* index was defined as ≥1 purchase within 6 months before admission, while medication use *after* index was defined as ≥1 purchase within 4 months after discharge (assessed among patients alive 1 week after discharge).

For contextualization of the mortality risk and hospital admission, data on the annual mortality per 100 inhabitants and inpatient admissions in the overall Swedish population during calendar years 2012 and 2013 were obtained through publicly available records at Statistics Sweden and the National Board of Health and Welfare, respectively.^17,18^

### Missing data

Of 23,003 patients with non-cardioembolic AIS, 11,358 (49.4%) had missing NIHSS scores and were excluded. No TIA patients lacked ABCD^2^ scores. The characteristics of patients with missing NIHSS scores were similar to those with moderate/severe AIS (Supplemental Material III), and their risk of all-cause mortality was similar to that among those with moderate AIS (Supplemental Material IV). Excepting NIHSS scores, proportions of missing data in baseline variables were low (less than 3%). The participation rate in the 3-month follow-up questionnaire, for patients with non-cardioembolic AIS, was 85.4%; similar to the participation rate for all AIS patients registered in Riksstroke during 2013 (86%).^19^

### Statistical analysis

Baseline characteristics, medication use, and re-admissions were assessed using descriptive statistics. Cumulative incidence of death at 30, 90, and 365 days after index event were estimated with the Kaplan-Meier method. Mortality rates were calculated as deaths per 100 person-years. We used Cox proportional hazards regression to estimate hazard ratios (HRs) and 95% confidence intervals (CIs) for all-cause mortality until January 31, 2014. HRs were adjusted for variables based on their potential clinical relevance; the model included age (continuously), sex, vascular risk factors and cancer. All analyses were stratified by age at index (40–64; 65–74; 75–84; 85–100 years). Degree of disability at 3 months were transformed into estimated modified Rankin Scale (mRS) scores using a previously validated translation^20^ and dichotomized as 0–2 (no–slight disability) or 3–5 (moderate–severe disability).

To assess the generalizability of the results, analyses were also performed among patients with non-cardioembolic TIA or AIS, without risk or severity scores cut-offs, i.e. encompassing ABCD^2^ 0–6 and NIHSS 0–42.

Data management and statistical analyses were performed using SAS/STAT® version 9.4 (SAS Institute, Inc.).

## Results

During 2012–2013, 47,702 patients (40–100 years) were registered in Riksstroke with TIA or AIS. After application of eligibility criteria, 10,053 patients (mean age 72.6 years) with high-risk TIA (n = 1,645) or minor AIS (n = 8,408) were included in the study (Supplemental Material I). Mean and median follow-up time was 1.9 years. Mean ABCD^2^ score was 6.1 (high-risk TIA), and mean NIHSS score was 1.8 (minor AIS), without substantial difference between age groups. In total, 45.2% were women, fewer in the youngest age group (35.4%) than among the oldest (62.9%). With advancing age, most vascular risk factors increased, except smoking, which decreased with increasing age, and diabetes, which was more prevalent among patients aged 65–74 years (27.9%) and 75–84 years (25.2%) than among younger or older patients. Of all patients, 31.9% used antiplatelets before index, and 95.5% after discharge. Among antiplatelet users, <10% used more than 1 antiplatelet before index (Table 1), and nearly 20% after discharge (Table 2). Antihypertensive and statin use increased after discharge, whereas use of glucose-lowering drugs remained mostly unaltered (Table 2).

**Table 1.** Baseline characteristics in 10,053 patients with high-risk TIA or minor ischemic stroke, overall and by age.

	All n = 10,053	40-64 yrsn = 2,339	65-74 yrsn = 3,111	75-84 yrsn = 2,988	85-100 yrsn = 1,615
High-risk TIA	1645 (16.4)	205 (8.8)	557 (17.9)	564 (18.9)	319 (19.8)
Mean age, yrs (SD)	72.6 (11.5)	56.8 (6.2)	69.6 (2.8)	79.4 (2.8)	88.8 (3.1)
Female sex	4541 (45.2)	827 (35.4)	1201 (38.6)	1497 (50.1)	1016 (62.9)
Vascular risk factors					
Previous stroke or TIA	1546 (15.4)	184 (7.9)	457 (14.7)	572 (19.1)	333 (20.6)
Hypertension^a^	3548 (35.3)	564 (24.1)	1147 (36.9)	1231 (41.2)	606 (37.5)
Diabetes	2384 (23.7)	473 (20.2)	867 (27.9)	753 (25.2)	291 (18.0)
Ischemic heart disease	1227 (12.2)	110 (4.7)	347 (11.2)	473 (15.8)	297 (18.4)
Smoking	1722 (17.1)	753 (32.2)	666 (21.4)	249 (8.3)	54 (3.3)
Cancer	600 (6.0)	62 (2.7)	184 (5.9)	238 (8.0)	116 (7.2)
Drugs before admission					
Antiplatelets	3207 (31.9)	323 (13.8)	911 (29.3)	1234 (41.3)	739 (45.8)
Aspirin	3034 (30.2)	304 (13.0)	859 (27.6)	1175 (39.3)	696 (43.1)
Clopidogrel	284 (2.8)	36 (1.5)	89 (2.9)	109 (3.6)	50 (3.1)
Other P2Y12-I	23 (0.2)	5 (0.2)	10 (0.3)	6 (0.2)	2 (0.1)
Dipyridamole	136 (1.4)	12 (0.5)	44 (1.4)	52 (1.7)	28 (1.7)
Antiplatelet regimen^b^					
Single	2946 (91.9)	290 (89.8)	825 (90.6)	1129 (91.5)	702 (95.0)
Dual	252 (7.9)	32 (9.9)	81 (8.9)	102 (8.3)	37 (5.0)
Antihypertensives	5850 (58.2)	955 (40.8)	1806 (58.1)	2000 (66.9)	1089 (67.4)
Glucose-lowering drugs	1724 (17.1)	334 (14.3)	646 (20.8)	546 (18.3)	198 (12.3)
Statins	2404 (23.9)	393 (16.8)	859 (27.6)	871 (29.1)	281 (17.4)

Note: Data presented as n (%) unless otherwise specified.

TIA: transient ischemic attack; SD: standard deviation; P2Y12-I: P2Y12-inhibitor.

^a^Prescription of ≥2 different classes of antihypertensives before index.

^b^Denominator includes patients with ≥1 antiplatelet (9 patients with ≥3 antiplatelets).

**Table 2.** Medication use after high-risk TIA or minor ischemic stroke in 9,955 patients surviving discharge with 1 week.

Drugs after discharge	Alln = 9,955	40-64 yrsn = 2,331	65-74 yrsn = 3,098	75-84 yrsn = 2,955	85-100 yrsn = 1,571
Antiplatelets	9508 (95.5)	2263 (97.1)	2975 (96.0)	2814 (95.2)	1456 (92.7)
Aspirin	5849 (58.8)	1348 (57.8)	1755 (56.6)	1764 (59.7)	982 (62.5)
Clopidogrel	4524 (45.4)	1125 (48.3)	1510 (48.7)	1321 (44.7)	568 (36.2)
Other P2Y12-I	33 (0.3)	7 (0.3)	15 (0.5)	7 (0.2)	4 (0.3)
Dipyridamole	1093 (11.0)	265 (11.4)	368 (11.9)	337 (11.4)	123 (7.8)
Antiplatelet regimen^a^					
Single	7634 (80.3)	1816 (80.2)	2347 (78.9)	2231 (79.3)	1240 (85.2)
Dual	1758 (18.5)	412 (18.2)	583 (19.6)	552 (19.6)	211 (14.5)
Antihypertensives	7632 (76.7)	1612 (69.2)	2449 (79.1)	2388 (80.8)	1183 (75.3)
Glucose-lowering drugs	1838 (18.5)	394 (16.9)	700 (22.6)	551 (18.6)	193 (12.3)
Statins	7843 (78.8)	2019 (86.6)	2678 (86.4)	2357 (79.8)	789 (50.2)

Note: Data presented as n (%).

P2Y12-I: P2Y12-inhibitor.

^a^Denominator includes patients with ≥1 antiplatelet (116 patients with ≥3 antiplatelets).

Among patients with minor AIS, 3.6% (n = 306) were ADL-dependent on admission (age 40–64: 0.7%; age 85–100: 12.1%). Among minor AIS patients with available mRS scores at 3 months after stroke (n = 6,927), patients with moderate–severe disability (mRS 3–5) were on average older, had more prior cerebrovascular events, and had a higher prevalence of comorbidities and medication use before index than patients with no–slight disability (mRS 0–2) (Supplemental Material V). Except for lesser use of statins among patients with moderate-severe disability, medication use after discharge did not vary with degree of disability (Supplemental Material VI).

### Length of index hospital stay and re-admissions

Mean length of stay (LOS) for the index hospitalization was 9.2 days (median 5.0) for patients discharged alive (age 40–64: 8.6 days; age 65–74: 8.4 days; age 75–84: 9.4 days; age 85–100: 11.0 days). For AIS patients who died during index hospitalization (n = 84), mean LOS was 16.2 days (median 13.5). Most AIS patients were discharged to home (78.3%), the number of patients discharged to inpatient rehabilitation increased with age (age 40–64: 9.4%; age 85–100: 15.4%). For AIS patients with no–slight post-stroke disability at 3 months, mean LOS for the index hospitalization was 6.9 days (median 5.0) and 91.4% were discharged to home; corresponding results for patients with moderate–severe disability were 14.4 days (median 10.0) and 65.0%.

During the first 30 days after index discharge, approximately 10% of patients in each age group had ≥1 re-admission (Table 3). When analyzing re-admissions during the first year, the difference between the youngest and oldest was more pronounced; 29.2% of patients 40–64 years had ≥1 re-admission compared with 47.2% in the oldest age group. In comparison, on average 13% of the entire Swedish population (≥40 years) had ≥1 inpatient admission per year; 8% in 40–64 year-olds and 41% in those aged ≥85 years.^18^

**Table 3.** All-cause re-admissions in 9,969 patients discharged alive after high-risk TIA or minor ischemic stroke.

	Alln = 9,969	40-64 yrsn = 2,336	65-74 yrsn = 3,100	75-84 yrsn = 2,957	85-100 yrsn = 1,576
Re-admissions, number of patients (%)					
Day 1–30	965 (9.7)	196 (8.4)	283 (9.1)	312 (10.6)	174 (11.0)
Day 1–90	1743 (17.5)	346 (14.8)	489 (15.8)	560 (18.9)	348 (22.1)
Day 1–365	3642 (36.5)	683 (29.2)	1034 (33.4)	1181 (39.9)	744 (47.2)

For AIS patients with no–slight post-stroke disability, 6.3% had ≥1 re-admission within the first 30 days and 27.1% within the first year (Supplemental Material VII). These percentages were almost twice as high (10.7% within 30 days and 45.7% within 1 year) for AIS patients with moderate–severe disability.

### Mortality

**Figure 1.**
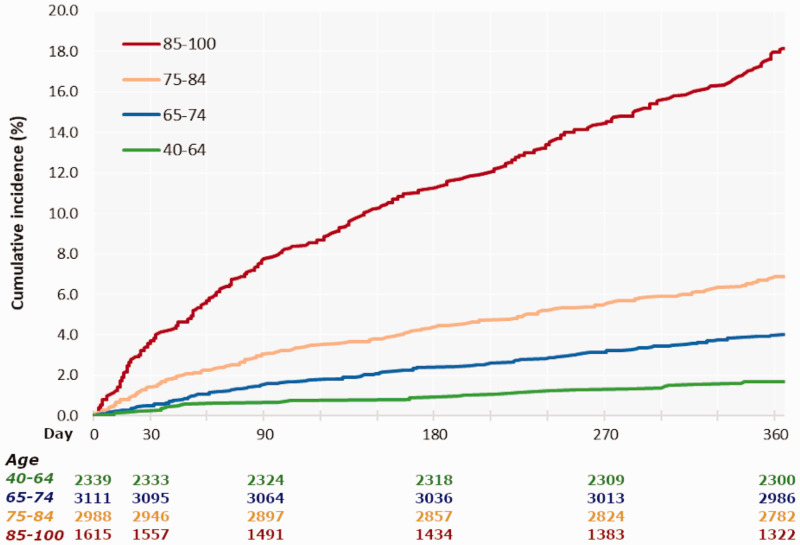
Cumulative incidence of all-cause mortality in 10,053 patients with high-risk TIA or minor ischemic stroke, by age. Numbers below graph denote patients alive at each time point.

The risk of all-cause death was 1.2% (95% CI 1.0–1.5) at 30 days, 2.8% (2.5–3.1) at 90 days and 6.6% (6.1–7.1) at 365 days in the overall population. The age groups differed substantially, with higher risk in patients aged 75–84 years, and highest in the oldest age group, compared to the youngest (Figure 1). The 1-year risk of mortality in patients with high-risk TIA or minor AIS was generally higher as compared to the annual mortality in the overall population in Sweden, as reported by Statistics Sweden^17^ (Supplemental Material VIII).

The rate of all-cause death per 100 person-years was 15.2 (95% CI 12.8–18.2) within 0–30 days, 9.5 (8.1–11.1) within 31–90 days, and 6.9 (6.4–7.4) within 0–365 days after index admission. For all age groups, the mortality rate was highest close to the index event. Yet, the 30-day mortality rate was 15 times higher in the oldest age group compared with the youngest (Table 4).

**Table 4.** Mortality rate per 100 person-years (95% CI) in 10,053 patients during the first year after high-risk TIA or minor ischemic stroke, by age.

Days after index	Alln = 10,053		40-64 yrsn = 2,339		65-74 yrsn = 3,111		75-84 yrsn = 2,988		85-100 yrsn = 1,615	
	Deaths	Rate	Deaths	Rate	Deaths	Rate	Deaths	Rate	Deaths	Rate
0–30	125	15.2 (12.8–18.2)	6	3.1 (1.1–6.8)	16	6.3 (3.8–10.2)	43	17.6 (13.1–23.8)	60	46.0 (35.8–59.3)
31–90	154	9.5 (8.1–11.1)	9	2.4 (1.2–4.5)	32	6.3 (4.5–8.9)	38	10.0 (7.5–13.3)	65	25.9 (20.3–33.1)
0–365	663	6.9 (6.4–7.4)	39	1.7 (1.2–2.3)	125	4.1 (3.4–4.9)	206	7.2 (6.3–8.2)	293	20.3 (18.1–22.8)

Age was a strong risk factor for death, both in unadjusted and adjusted analyses (Table 5). This association was also observed within each age strata; for each year of increased age, the adjusted HR of death was 1.04 (95% CI 0.99–1.08) in the youngest age group, and 1.12 (1.09–1.15) in the oldest (Supplemental Material IX). Diabetes, ischemic heart disease, cancer, smoking, and male sex was associated with an increased risk of death in the adjusted analyses; no statistically significant associations between death and previous TIA, previous stroke, or prior use of antihypertensives were observed (Table 5).

**Table 5.** Unadjusted and adjusted hazard ratios (HR) and 95% CI for all-cause mortality in 10,053 patients with high-risk TIA or minor ischemic stroke.

	Unadjusted		Age- & sex adjusted		Adjusted^a^	
	HR	95% CI	HR	95% CI	HR	95% CI
Age (group)						
65–74	2.28	1.76–2.96	2.29	1.77–2.97	2.18	1.71–2.89
75–84	3.97	3.10–5.09	4.08	3.19–5.23	3.94	2.97–4.96
≥85	11.55	9.08–14.71	12.16	9.53–15.52	12.00	8.42–14.12
Male sex	0.86	0.77–0.97	1.20	1.07–1.35	1.12	1.00–1.26
Previous stroke	1.44	1.21–1.71	1.17	0.99–1.40	1.08	0.91–1.28
Previous TIA	1.20	0.98–1.47	0.97	0.79–1.18	0.95	0.77–1.16
Hypertension	1.23	1.09–1.38	1.11	0.98–1.24	0.97	0.85–1.09
Diabetes	1.36	1.20–1.54	1.50	1.32–1.70	1.45	1.27–1.65
Ischemic heart disease	1.88	1.63–2.17	1.42	1.23–1.64	1.30	1.11–1.51
Active smoking	0.73	0.62–0.87	1.39	1.16–1.66	1.42	1.18–1.70
Cancer	2.80	2.37–3.31	2.29	1.94–2.71	2.19	1.84–2.59

^a^All variables included.

### Any severity population

When including low-risk TIA or moderate/severe AIS, analyses revealed a lower proportion of AIS and of diabetes than in the main study population. No major deviations was seen in medications and re-admission patterns, but mortality rate was higher, particularly close to the index event and in older patients (Supplemental Materials X-XIV).

## Discussion

### Main findings

This study including real-world patients with high-risk TIA or minor ischemic stroke demonstrated a high prevalence of vascular risk factors and high use of health-care resources in terms of index LOS and subsequent re-admissions. Further, we observed low mortality during index hospitalization, yet high mortality rate after discharge, particularly shortly after the event. Naturally, there was a strong association between advancing age and increased risk of death. Still, the 1-year risk of mortality in patients with high-risk TIA or minor AIS was generally higher as compared to the mortality in the overall population in Sweden,^17^ suggesting an excess mortality in this patient population.

Ischemic events are rarely isolated phenomena, illustrated by the large proportion of patients (32%) already on antiplatelet medications before the index event. Established risk factors for cerebrovascular disease, such as smoking, diabetes mellitus and hypertension were common. Yet, the prevalence of hypertension in this study (35%) was much lower than in previous epidemiological studies,^4,6,7^ and recent DAPT-trials^9–11^; e.g. in THALES >76% of the participants had hypertension. This is probably due to the more restrictive definition used in this study (prescription of ≥2 different classes of antihypertensives before index), which was chosen as clinical data on hypertension were unavailable.^16^

Comparing medication use before and after the index event, there was an expected increase in antiplatelet therapy (from 32% to 96%). Even though at the time not recommended in guidelines, we also observed an increase in the percentage of antiplatelet users receiving DAPT (from 8% to 19%). With increasing support for DAPT from recent trials,^9–11^ the proportion of patients treated with DAPT may be larger in the future. Still, according to the Swedish Stroke Register (2019), 18% of TIA patients and 23% of stroke patients (both without atrial fibrillation) who were prescribed antiplatelet therapy at discharge received DAPT.^21^ The proportion of patients prescribed antihypertensives increased from 58% to 77%, and statins from 24% to 79%, indicating hypertension and dyslipidemia were identified at index and not before. Interestingly, an increase was not observed for glucose-lowering drugs (17% before and 18% after).

After the index event, about 10% of patients in each age group had ≥1 re-admission within the first month. Overall, 36% had ≥1 re-admission within the first year, with a pronounced difference by age; 29% in 40–64 year-olds and 47% in 85–100 year-olds. In comparison, publicly available data on inpatient care demonstrate that on average 13% of the entire Swedish population (≥40 years) had ≥1 inpatient admission per year; 8% in 40–64 year-olds and 41% in those aged ≥85 years.^18^ This suggests a higher healthcare resource use in the high-risk TIA and minor AIS population, despite by definition presenting with relatively mild symptoms. A recently published study of Swedish patients with ischemic stroke of any severity and etiology (mean age 78 years) points out an association between burden of comorbidity and re-admissions, and reports a higher proportion of re-admissions (44%) than in our study.^5^ This difference could be explained by lower prevalence of comorbidities and lower mean age (73 years) in our study population.

The overall mortality rate (6.9 deaths per 100 person-years within 1 year) was similar to previously reported rates for combined minor stroke/TIA populations,^14,15^ all-severity AIS,^8,22^ minor AIS alone,^15^ and TIA alone.^15,22^ The mortality rate was highest shortly after the event for all age categories, and particularly high for older patients.

In AIS-patients with moderate-severe post-stroke disability, re-admission within the first year was nearly twice as common as in patients with no-slight disability. This could be attributed to higher levels of premorbid disability, and greater stroke severity requiring longer inpatient rehabilitation. It is possible that patients with worse long-term functional outcomes also have a higher prevalence of short-term stroke-related complications, contributing to the higher occurrence of re-admissions.

Risk factors for all-cause mortality showed a substantial influence by increasing age, with a dramatically increased risk of death among the oldest. With increasing age, outcomes worsened not just in terms of mortality, but also in terms of discharge disposition and subsequent inpatient admissions. The proportion of AIS patients discharged to inpatient rehabilitation facilities also increased with age, presumably because younger patients are more likely to remain functionally independent after the event and therefore more often are referred to outpatient or ambulatory services. Indeed, patients with no-slight disability were younger than those with moderate-severe post-stroke disability.

This observational study represents real-world TIA/stroke patients from all stroke units in Sweden, covering approximately 75% of TIA and 95% of AIS in the country.^19,23^ Riksstroke have low levels of missing data,^23^ and the national registries used in this study have comprehensive data from all Swedish citizens.

Some shortcomings are however worth mentioning: important variables were unavailable, e.g. blood pressure and subsequent events during, and shortly after, the index hospitalization. In addition to the clinical challenge of distinguishing early recurrence from the index event itself, it is not captured by Riksstroke and NPR; thus, the important outcome of subsequent stroke had to be omitted. Further, registration of dates may differ between Riksstroke and NPR, necessitating a wash-out period when counting re-admissions. NIHSS is strongly predictive of outcome after stroke^24^ and the high proportion of missing data in our study was unfortunate, but reflects the nature of this all-comer, all-inclusive national stroke registry. In addition, full similarity with recent DAPT trial populations was not possible. Some trials define high-risk TIA by ABCD^2^ score of ≥4p,^9,10,25^ while others use ≥6p^11^; minor AIS is in some studies defined by NIHSS score of ≤3p,^9,10^ and in others by ≤5p. We used the same inclusion criteria as THALES, but lacked data to enable application of all THALES exclusion criteria, e.g. liver disease or planned carotid surgery.

## Conclusions

Our study contributes to the understanding of the characteristics and challenges for healthcare in this TIA/stroke population assumed eligible for DAPT. While theoretically representing a subset of TIA/stroke patients with no or relatively mild injury, the burden of vascular risk factors and mortality was substantial. In addition, their all-cause re-admission rate was high, indicating a high healthcare resource use and potential unmet needs.

## Supplemental Material

sj-pdf-1-eso-10.1177_2396987320975980 - Supplemental material for Age in relation to comorbidity and outcome in patients with high-risk TIA or minor ischemic stroke: A Swedish national observational study Click here for additional data file. Supplemental material, sj-pdf-1-eso-10.1177_2396987320975980 for Age in relation to comorbidity and outcome in patients with high-risk TIA or minor ischemic stroke: A Swedish national observational study by Oskar Fasth, Eva Lesén, Peter Appelros, Bahman Farahmand, Jonatan Hedberg, Per Ladenvall, Carl Mellström and Signild Åsberg in European Stroke Journal
