# Supplementary material for: Age in relation to comorbidity and outcome in patients with high-risk TIA or minor ischemic stroke: A Swedish national observational study
Source: Eur Stroke J. 2020 Dec 13;6(1):53–61. doi: 10.1177/2396987320975980 (PMC7995324; doi:10.1177/2396987320975980)
Supplement: sj-pdf-1-eso-10.1177_2396987320975980 - Supplemental material for Age in relation to comorbidity and outcome in patients with high-risk TIA or minor ischemic stroke: A Swedish national observational study [file sj-pdf-1-eso-10.1177_2396987320975980.pdf]

Supplemental material

Age in relation to comorbidity and outcome in patients with high-risk TIA or minor ischemic stroke: a Swedish national observational study

Authors

Fasth, Oskar MD[1]; Lesén, Eva PhD[2]; Appelros, Peter MD, PhD[3]; Farahmand, Bahman Bsc, PhD[4]; Hedberg, Jonatan MSc[2]; Ladenvall, Per MD, PhD[2]; Mellström, Carl PhL[2]; Åsberg, Signild MD, PhD[1]

[1]Department of Neuroscience, Uppsala University, Sweden

[2]AstraZeneca AB, Göteborg, Sweden

[3]University Health Care Research Center, Örebro University, Örebro, Sweden

[4]Epi-Consultant, Stockholm, Sweden

Table of contents

Table of contents 2
Supplemental Material I. Selection of study population..3
Supplemental Material II. List of definitions and data sources for diagnoses and medication use, including ICD and ATC-codes...4
Supplemental Material III. Baseline characteristics of patients with non-cardioembolic ischemic stroke by stroke severity...6
Supplemental Material IV. Cumulative incidence of all-cause mortality in patients with non-cardioembolic ischemic stroke by stroke severity..7
Supplemental Material V. Baseline characteristics in 6,927 patients with minor ischemic stroke, by mRS at 3 months 8
Supplemental Material VI. Medication use after minor ischemic stroke in 6,927 patients, by mRS at 3 months 9
Supplemental Material VII. All-cause re-admissions after minor ischemic stroke in 6,927 patients, by mRS at 3 months...10
Supplemental Material VIII. 1-year risk of all-cause mortality in 10,053 patients with high-risk TIA or minor ischemic stroke, and annual mortality per 100 inhabitants in the overall Swedish population...11
Supplemental Material IX. Unadjusted and adjusted hazard ratios (HR) and 95% CI for all-cause mortality in 10,053 patients with high-risk TIA or minor ischemic stroke, by age....12
Supplemental Material X. Baseline characteristics in 21,702 patients with non-cardioembolic TIA or ischemic stroke of any severity, by age...13
Supplemental Material XI. Medication use after non-cardioembolic high-risk TIA or stroke of any severity in 21,180 patients surviving discharge with 1 week, by age......................14
Supplemental Material XII. All-cause re-admissions in 21,257 patients discharged alive after non-cardioembolic TIA or ischemic stroke of any severity, by age...........................15
Supplemental Material XIII. Cumulative incidence of all-cause mortality in 21,702 patients with non-cardioembolic TIA or ischemic stroke, by age..16
Supplemental Material XIV. Mortality rate per 100 person-years in 21,702 patients during the first year after non-cardioembolic TIA or ischemic stroke of any severity, by age 17

Supplemental Material I. Selection of study population

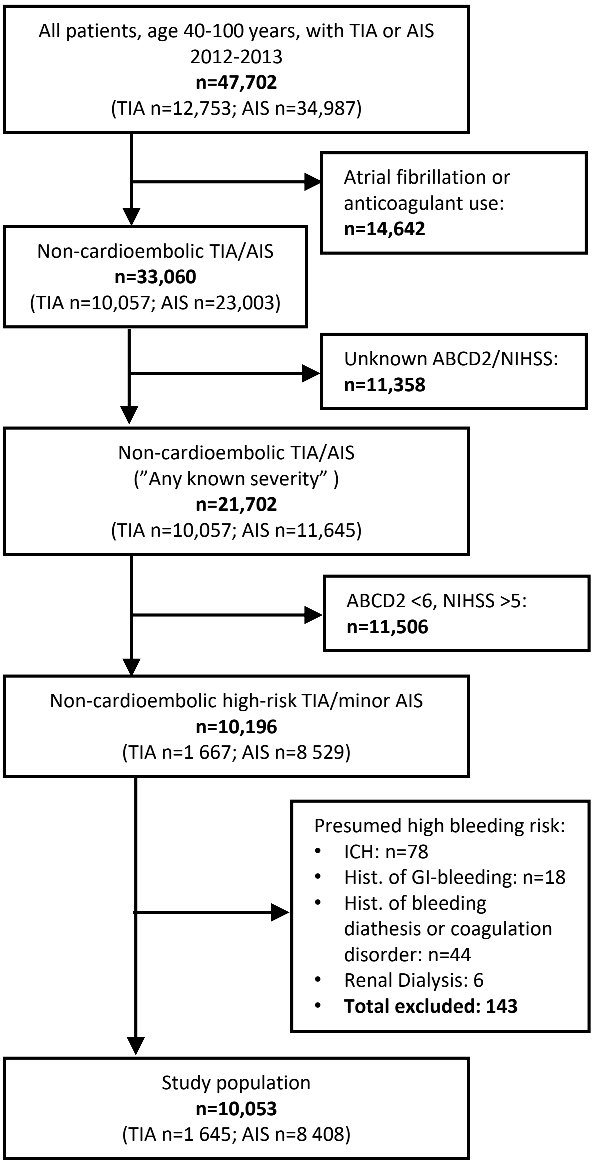

Supplemental Material II. List of definitions and data sources for diagnoses and medication use, including ICD and ATC-codes.

Diagnosis/medication	*Definition*	*Data source*
**Eligibility criteria**		
Acute ischemic stroke (AIS)	ICD-10: I63 – I64 (January 2012-December 2013)	RS
Transient ischemic attack (TIA)	ICD-10: G45.0-3, 8-9 (January 2012-and December 2013)	RS
Atrial fibrillation	ICD-10: I48 (any time prior to index)	RS, NPR
Oral anticoagulants	B01AA, B01AE07, B01AF, B01AX06 (in PDR within 6 months prior to index, or in RS on admission/discharge)	PDR, RS
Intracranial haemorrhage	ICD-10: I60-62, S06.4-6 (in NPR any time prior to index) and/or ICD-10: I61 (RS)	NPR, RS
Gastrointestinal bleeding	ICD-10: I85.0, K22.6, K25-28, K29.0, K92.0-2 (within 6 months prior to index)	NPR
Bleeding diathesis or coagulation disorder	ICD-10: D66, D67, D68.0, D68.1, D68.2, D68.3, D68.4, D68.8, D68.9, D69.1, D69.3, D69.4, D69.5, D69.6 (any time prior to index)	NPR
Renal dialysis	ICD-10: Z49.1, Z99.2 (within 6 months prior to index)	NPR
**Risk factors and comorbidities**		
Previous stroke or TIA	Previous stroke: ICD-10: I61 or I63-64 (in NPR any time prior to index) or registered in RS<br>Previous TIA: As registered in RS	NPR, RS
Hypertension	Purchase of ≥2 antihypertensive medication classes (see below) within 6 months prior to index	PDR
Diabetes mellitus	ICD-10: E10-E14 (in NPR any time prior to index), purchase of any antidiabetic (ATC A10A-B in PDR any time prior to index) or as registered in RS	NPR, PDR, RS
Ischemic heart disease	ICD-10: I20-25 (any time prior to index)	NPR
Active smoking	Registered in RS at index admission or at 3-month follow-up	RS
Cancer	ICD-10 C00-99 (within 3 years prior to index)	NPR
**Medications***		

**Antiplatelets**	*Any of the following ATC-codes:*	
ASA	B01AC06	PDR
Clopidogrel	B01AC04	PDR
Other P2Y12-inhibitors	B01AC22, B01AC24	PDR
Dipyridamole	B01AC07	PDR
**Any antihypertensive**	*Any of the following ATC-codes:*	
β-blockers	C07	PDR
Calcium channel blockers	C07F, C08, C09BB, C09DB	PDR
Non-loop diuretics	C02DA, C02L, C03A, C03B, C03D, C03E, C03X, C07C, C07D, C08G, C09BA, C09DA, C09XA52	PDR
α-adrenergic blockers	C02A, C02B, C02C	PDR
Vasodilators	C02DB, C02DD, C02DG, C04, C05	PDR
Renin-angiotensin system inhibitors	C09	PDR
**Glucose-lowering drugs**	A10A-B	PDR
Insulin	A10A	PDR
Oral antidiabetics	A10B	PDR
**Statins**	C10AA	PDR

RS: Riksstroke, NPR: National Patient Register, PDR: Prescribed Drugs Register

* Medication use prior to index defined as least one purchase within 180 days prior to index; medication use after discharge defined as at least one purchase within 4 months from discharge date in patients surviving index discharge with 7 days

Supplemental Material III. Baseline characteristics of patients with non-cardioembolic ischemic stroke by stroke severity

	Minor (N=8,529)	Moderate (N=1,660)	Severe (N=1,456)	Missing (N=11,358)
Mean age, yrs (SD)	72.1 (11.8)	74.8 (12.2)	76.0 (12.2)	74.7 (12.0)
Female sex	3,774 (44.2)	780 (47.0)	783 (53.8)	5 565 (49.0)
Vascular risk factors				
Previous stroke or TIA	1223 (14.3)	308 (18.6)	205 (14.1)	1826 (16.1)
Hypertension*	2951 (34.6)	571 (34.4)	544 (37.4)	4 154 (36.6)
Diabetes	1812 (21.2)	347 (20.9)	306 (21.0)	2 583 (22.7)
Ischemic heart disease	986 (11.6)	265 (16.0)	238 (16.3)	1 700 (15.0)
Smoking	1577 (18.5)	295 (17.8)	214 (14.7)	1 794 (15.8)
Cancer	506 (5.9)	106 (6.4)	91 (6.3)	821 (7.2)
Drugs before admission				
Antiplatelets	2588 (30.3)	606 (36.5)	497 (34.1)	3 997 (35.2)
Aspirin	2445 (28.7)	565 (34.0)	475 (32.6)	3 800 (33.5)
Clopidogrel	221 (2.6)	64 (3.9)	39 (2.7)	367 (3.2)
Other P2Y12-I	19 (0.2)	3 (0.2)	2 (0.1)	50 (0.4)
Dipyridamole	112 (1.3)	28 (1.7)	14 (1.0)	140 (1.2)
Antiplatelet regimen**				
Single	2385 (92.2)	554 (91.4)	464 (93.4)	3 651 (91.3)
Dual	197 (7.6)	50 (8.3)	33 (6.6)	332 (8.3)
Antihypertensives	4863 (57.0)	956 (57.6)	882 (60.6)	6 898 (60.7)
Glucose-lowering drugs	1289 (15.1)	244 (14.7)	211 (14.5)	1 921 (16.9)
Statins	1 950 (22.9)	363 (21.9)	331 (22.7)	2 568 (22.6)

Stroke severity categorized according to National Institutes of Health Stroke Scale (NIHSS) scores: Minor NIHSS ≤5, Moderate NIHSS 6-10, Severe NIHSS ≥11, Missing NIHSS.

Data are presented as n (%) unless otherwise specified.

TIA=Transient ischemic attack; SD=Standard deviation; P2Y12-I=P2Y12-inhibitor.

* Defined as prescription of ≥2 different classes of antihypertensive medications prior to index.

** Denominator includes patients with ≥1 antiplatelet (number of patients with ≥3 antiplatelets by stroke severity: Missing: 6, Moderate: 2, Severe: 0, Missing: 14)

Supplemental Material IV. Cumulative incidence of all-cause mortality in patients with non-cardioembolic ischemic stroke by stroke severity

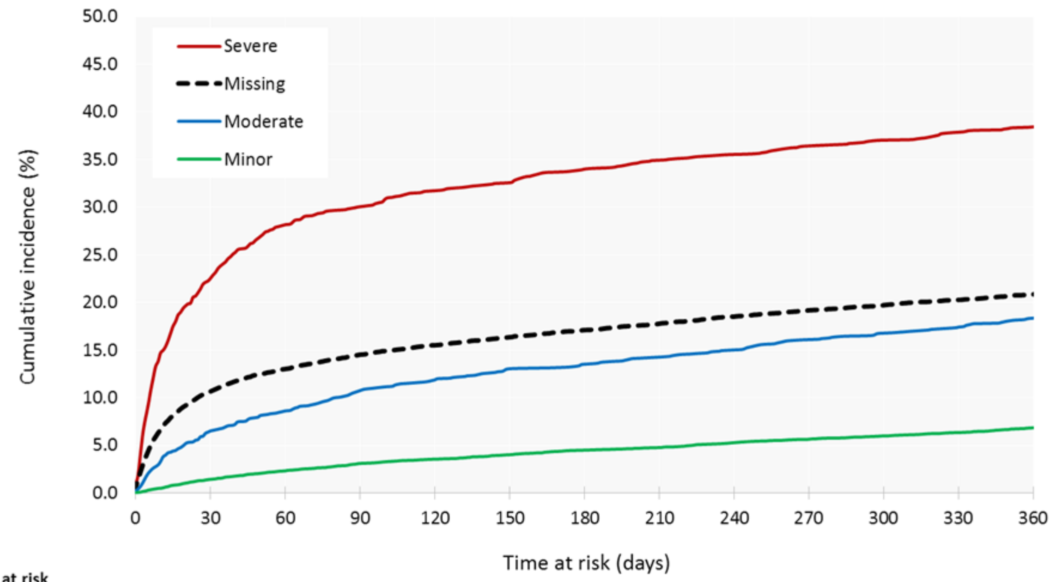

**Number at risk**						
Minor	8 529	8 410	8 267	8 147	8 050	7 942
Moderate	1 660	1 554	1 483	1 436	1 393	1 354
Severe	1 456	1 132	1 019	962	926	893
Missing	11 358	10 163	9 714	9 418	9 182	8 985

Stroke severity categorized according to National Institutes of Health Stroke Scale (NIHSS) scores: Minor NIHSS ≤5, Moderate NIHSS 6-10, Severe NIHSS ≥11, or Missing NIHSS.

Supplemental Material V. Baseline characteristics in 6,927 patients with minor ischemic stroke, by mRS at 3 months

	mRS 0-2 (N=4,374)	mRS 3-5 (N=2,553)
Mean age, yrs (SD)	68.7 (10.3)	77.3 (11.0)
Female sex	1628 (37.2)	1394 (54.6)
Vascular risk factors		
Previous stroke or TIA	513 (11.7)	448 (17.5)
Hypertension*	1447 (33.1)	999 (39.1)
Diabetes	823 (18.8)	619 (24.3)
Ischemic heart disease	393 (9.0)	365 (14.3)
Smoking	849 (19.4)	390 (15.3)
Cancer	213 (4.9)	171 (6.7)
Drugs before admission		
Antiplatelets	1143 (26.1)	949 (37.2)
Aspirin	1079 (24.7)	894 (35.0)
Clopidogrel	108 (2.5)	64 (2.5)
Other P2Y12-I	7 (0.2)	9 (0.4)
Dipyridamole	46 (1.1)	48 (1.9)
Antiplatelet regimen**		
Single	1049 (91.8)	884 (93.2)
Dual	91 (8.0)	64 (6.7)
Antihypertensives	2360 (54.0)	1628 (63.8)
Glucose-lowering drugs	581 (13.3)	446 (17.5)
Statins	1020 (23.3)	594 (23.3)

Data are presented as n (%) unless otherwise specified.

Patients with missing mRS at the 3-months follow-up were excluded

TIA=Transient ischemic attack; SD=Standard deviation; P2Y12-I=P2Y12-inhibitor.

* Defined as prescription of ≥2 different classes of antihypertensive medications prior to index.

**Denominator includes patients with ≥1 antiplatelet (4 patients in total had ≥3 antiplatelets)

Supplemental Material VI. Medication use after minor ischemic stroke in 6,927 patients, by mRS at 3 months

Drugs after discharge, n (%)	mRS 0-2 (N=4374)	mRS 3-5 (N=2553)
Antiplatelets	4272 (97.7)	2431 (95.2)
Aspirin	2517 (57.6)	1552 (60.8)
Clopidogrel	2139 (48.9)	1087 (42.6)
Other P2Y12-I	12 (0.3)	10 (0.4)
Dipyridamole	2018 (10.1)	496 (9.4)
Antiplatelet regimen*		
Single	3404 (79.7)	1986 (81.7)
Dual	817 (19.1)	426 (17.5)
Antihypertensives	3406 (77.9)	2031 (79.6)
Glucose-lowering drugs	672 (15.4)	479 (18.8)
Statins	3837 (87.7)	1846 (72.3)

Patients with missing mRS or who were dead at the 3-months follow-up were excluded.

P2Y12-I=P2Y12-inhibitor.

*Denominator includes patients with ≥1 antiplatelet (70 patients in total had ≥3 antiplatelets)

Supplemental Material VII. All-cause re-admissions after minor ischemic stroke in 6,927 patients, by mRS at 3 months

	mRS 0-2<br>n= 4,374	mRS 3-5<br>n=2,553
Patients with re-admissions, n (%)*		
Day 1-30	277 (6.3)	274 (10.7)
Day 1-90	498 (11.4)	534 (20.9)
Day 1-365	1185 (27.1)	1166 (45.7)

Patients with missing mRS or who were dead at the 3-months follow-up were excluded

Supplemental Material VIII. 1-year risk of all-cause mortality in 10,053 patients with high-risk TIA or minor ischemic stroke, and annual mortality per 100 inhabitants in the overall Swedish population

**High-risk TIA or minor ischemic stroke**		**Overall Swedish population****		
**Age (years)**	**1-year risk of mortality*, % (95% CI)**	**Age (years)**	**Mortality in 2012**	**Mortality in 2013**
40-64	1.7 (1.2-2.3)	40-44	0.1	0.1
		45-49	0.2	0.1
		50-54	0.3	0.3
		55-59	0.4	0.4
		60-64	0.7	0.7
65-74	4.0 (3.8-4.8)	65-69	1.1	1.1
		70-74	1.9	1.8
75-84	6.9 (6.0-7.8)	75-79	3.3	3.2
		80-84	6.2	6.0
85-100	18.1 (16.3-20.1)	85-89	11.8	11.2
		90+	23.9	23.3

* 1-year cumulative incidence of all-cause mortality after the index event, estimated with the Kaplan-Meier method

** Number of deaths per 100 Swedish inhabitants per calendar year (2012 and 2013, respectively). Available from:
http://www.statistikdatabasen.scb.se/pxweb/sv/ssd/START__BE__BE0101__BE0101I/Dodstal/

Supplemental Material IX. Unadjusted and adjusted hazard ratios (HR) and 95% CI for all-cause mortality in 10,053 patients with high-risk TIA or minor ischemic stroke, by age

	40-64 years (n=2,339)				65-74 years (n=3,111)				75-84 years (n=2,988)				85-100 years (n=1,615)			
	Crude		Adjusted*		Crude		Adjusted*		Crude		Adjusted*		Crude		Adjusted*	
	HR	95% CI	HR	95% CI	HR	95% CI	HR	95% CI	HR	95% CI	HR	95% CI	HR	95% CI	HR	95% CI
Age per year	1.06	1.02-1.11	1.04	0.99-1.08	1.07	1.02-1.13	1.06	1.01-1.11	1.10	1.06-1.14	1.11	1.07-1.15	1.11	1.08-1.14	1.12	1.09-1.15
Male sex	1.12	0.70-1.81	1.03	0.63-1.67	1.16	0.88-1.53	1.14	0.86-1.50	1.21	0.98-1.48	1.16	0.94-1.43	1.23	1.03-1.47	1.24	1.04-1.49
Previous stroke	1.50	0.60-3.71	1.78	0.70-4.52	1.67	1.16-2.42	1.53	1.00-2.11	0.94	0.68-1.30	0.87	0.62-1.20	1.18	0.92-1.51	1.12	0.86-1.44
Previous TIA	1.34	0.49-3.65	1.49	0.53-4.19	1.07	0.64-1.77	0.95	0.57-1.59	1.15	0.83-1.60	1.18	0.85-1.65	0.79	0.58-1.08	0.78	0.57-1.06
Hypertension	1.77	1.11-2.84	1.36	0.82-2.25	1.25	0.96-1.63	1.03	0.78-1.36	1.05	0.85-1.30	0.95	0.76-1.18	1.01	0.84-1.21	0.93	0.77-1.12
Diabetes	1.54	0.93-2.54	1.30	0.77-2.20	1.75	1.34-2.29	1.61	1.22-2.13	1.32	1.06-1.65	1.29	1.03-1.62	1.56	1.27-1.92	1.57	1.27-1.94
Ischemic heart disease	2.13	0.98-4.62	1.33	0.57-3.13	1.45	1.01-2.09	1.16	0.79-1.70	1.48	1.15-1.90	1.30	1.00-1.69	1.40	1.14-1.73	1.26	1.01-1.57
Smoker	1.01	0.62-1.65	1.09	0.67-1.79	1.57	1.18-2.10	1.76	1.31-2.36	1.52	1.11-2.10	1.71	1.24-2.36	1.19	0.76-1.86	1.38	0.87-2.17
Cancer	16.87	10.20-27.91	15.90	9.39-26.92	3.52	2.48-5.01	3.25	2.28-4.66	2.48	1.88-3.28	2.39	1.80-3.17	1.25	0.91-1.71	1.09	0.79-1.50

*=All variables included.

Supplemental Material X. Baseline characteristics in 21,702 patients with non-cardioembolic TIA or ischemic stroke of any severity, by age

	All<br>n=21,702	40-64 yrs<br>n=5,236	65-74 yrs<br>n=6,489	75-84 yrs<br>n=6,290	85-100 yrs<br>n=3,597
High-risk TIA	10057 (46.3)	2567 (48.2)	3149 (48.5)	2900 (46.1)	1441 (40.1)
Mean age, yrs (SD)	72.4 (11.9)	56.2 (6.3)	69.7 (2.8)	79.4 (2.9)	88.8 (3.1)
Female sex	10366 (47.8)	2094 (39.3)	2705 (41.7)	3269 (52.0)	2298 (63.9)
Vascular risk factors					
Previous stroke or TIA	3592 (16.6)	481 (9.0)	1038 (16.0)	1305 (20.7)	768 (21.4)
Hypertension*	7294 (33.6)	1128 (21.2)	2242 (34.6)	2553 (40.6)	1371 (38.1)
Diabetes	4061 (18.7)	824 (15.5)	1360 (21.0)	1303 (20.7)	574 (16.0)
Ischemic heart disease	2729 (12.6)	260 (4.9)	701 (10.8)	1044 (16.6)	724 (20.1)
Smoking	3313 (15.9)	1544 (29.8)	1182 (18.9)	468 (7.8)	119 (3.5)
Cancer	1324 (6.1)	151 (2.8)	394 (6.1)	524 (8.3)	255 (7.1)
Drugs before admission					
Antiplatelets	6970 (32.1)	733 (13.8)	1896 (29.2)	2640 (42.0)	1701 (47.3)
Aspirin	6531 (30.1)	673 (12.6)	1769 (27.3)	2481 (39.4)	1608 (44.7)
Clopidogrel	680 (3.1)	102 (1.9)	200 (3.1)	258 (4.1)	120 (3.3)
Other P2Y12-I	56 (0.3)	12 (0.2)	21 (0.3)	18 (0.3)	5 (0.1)
Dipyridamole	332 (1.5)	28 (0.5)	93 (1.4)	142 (2.3)	69 (1.9)
Antiplatelet regimen**					
Single	6358 (91.2)	653 (89.1)	1716 (90.5)	2388 (90.5)	1601 (94.1)
Dual	595 (8.5)	78 (10.2)	173 (9.1)	245 (9.3)	99 (5.8)
Antihypertensives	12 209 (56.3)	1991 (37.4)	3619 (55.8)	4176 (66.4)	2423 (67.4)
Glucose-lowering drugs	2934 (13.5)	579 (10.9)	1023 (15.8)	945 (15.0)	387 (10.8)
Statins	5173 (23.8)	838 (15.7)	1794 (27.6)	1899 (30.2)	642 (17.8)

Data are presented as n (%) unless otherwise specified.

TIA=Transient ischemic attack; SD=Standard deviation; P2Y12-I=P2Y12-inhibitor.

* Defined as prescription of ≥2 different classes of antihypertensive medications prior to index.

** Denominator includes patients with ≥1 antiplatelet (17 patients in total had ≥3 antiplatelets)

Supplemental Material XI. Medication use after non-cardioembolic high-risk TIA or stroke of any severity in 21,180 patients surviving discharge with 1 week, by age

Drugs after discharge, n (%)	All n=21,180	40-64 yrs n=5,286	65-74 yrs n=6,410	75-84 yrs n=6,128	85-100 yrs n=3,356
Antiplatelets	20003 (95.3)	5048 (95.5)	6158 (96.1)	5754 (93.9)	3043 (90.7)
Aspirin	12398 (58.5)	3048 (57.7)	3715 (58.0)	3587 (58.5)	2048 (61.0)
Clopidogrel	9415 (44.5)	2442 (46.2)	3043 (47.5)	2730 (44.5)	1200 (35.8)
Other P2Y12-I	92 (0.4)	19 (0.4)	35 (0.5)	28 (0.5)	10 (0.3)
Dipyridamole	2018 (9.5)	496 (9.4)	673 (10.5)	623 (10.2)	226 (6.7)
Antiplatelet regimen*					
Single	16327 (81.6)	4153 (82.3)	4937 (80.2)	4619 (80.3)	2618 (86.0)
Dual	3435 (17.2)	833 (16.5)	1136 (18.4)	1057 (18.4)	409 (13.4)
Antihypertensives	15110 (71.3)	3155 (59.7)	4770 (74.4)	4735 (77.3)	2450 (73.0)
Glucose-lowering drugs	3011 (14.2)	666 (12.6)	1086 (16.9)	916 (14.9)	343 (10.2)
Statins	16168 (76.3)	4404 (83.3)	5468 (85.3)	4756 (77.6)	1540 (45.9)

*Denominator includes patients with ≥1 antiplatelet (241 patients in total had ≥3 antiplatelets)

P2Y12-I=P2Y12-inhibitor.

Supplemental Material XII. All-cause re-admissions in 21,257 patients discharged alive after non-cardioembolic TIA or ischemic stroke of any severity, by age

	All<br>n=21,257	40-64 yrs<br>n=5,298	65-74 yrs<br>n=6,417	75-84 yrs<br>n=6,144290	85-100 yrs<br>n=3,398
Patients with re-admissions, n (%)*					
Day 1-30	2 056 (9.7)	462 (8.7)	573 (8.9)	641 (10.4)	380 (11.2)
Day 1-90	3 653 (17.2)	768 (14.5)	985 (15.3)	1 157 (18.8)	743 (21.9)
Day 1-365	7 519 (35.4)	1 473 (27.8)	2 057 (32.1)	2 421 (39.4)	1 568 (46.1)

Supplemental Material XIII. Cumulative incidence of all-cause mortality in 21,702 patients with non-cardioembolic TIA or ischemic stroke, by age

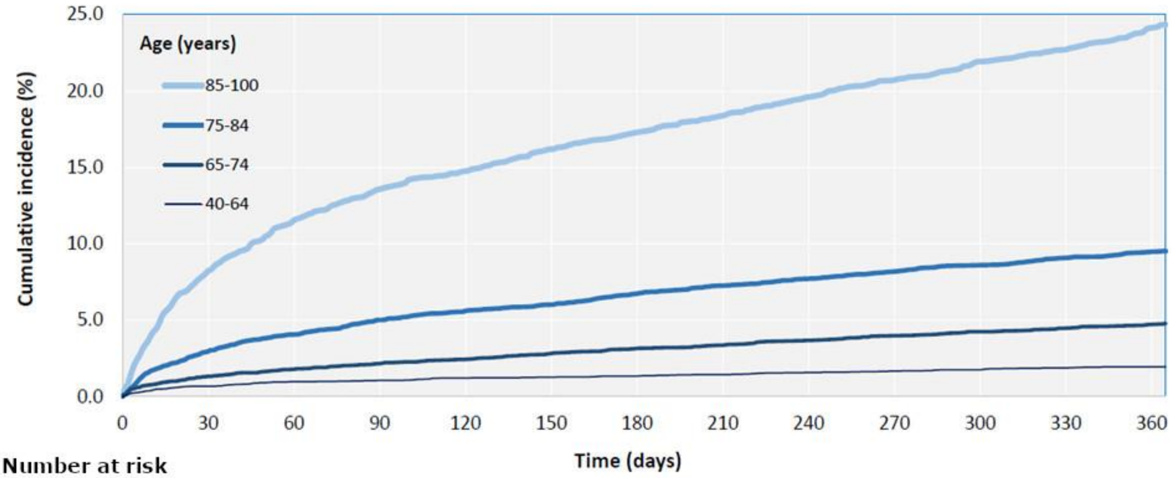

Number at risk

40-64	5326	5290	5270	5222
65-74	6489	6404	6350	6180
75-84	6290	6107	5975	5691
85-100	3597	3308	5691	2822

Supplemental Material XIV. Mortality rate per 100 person-years in 21,702 patients during the first year after non-cardioembolic TIA or ischemic stroke of any severity, by age

	All		40-64 years		65-74 years		75-84 years		85-100 years	
Days after index	Deaths	Rate	Deaths	Rate	Deaths	Rate	Deaths	Rate	Deaths	Rate
0-30	603	34.4 (31.8-37.2)	36	8.3 (6.0-11.5)	85	16.1 (13.0-19.9)	186	36.6 (31.7-42.3)	296	105.3 (93.9-118.0)
31-90	396	11.5 (10.5-12.7)	20	2.3 (1.5-3.6)	55	5.3 (4.0-6.8)	129	13.0 (10.9-15.4)	192	36.5 (31.7-42.1)
0-365	1 889	9.2 (8.8-9.7)	104	2.0 (1.6-2.4)	311	4.9 (4.4-5.5)	599	10.2 (9.4-11.0)	875	29.2 (27.3-31.2)

Mortality rate per 100 person-years (95% CI)
